# Human Research Protections during Emergencies: An Integrative Review

**DOI:** 10.1002/eahr.70009

**Published:** 2026-05-04

**Authors:** Lauren M. Sauer, Megan K. Singleton, Andrew Stolbach, Jonathan M. Links, Beth Resnick, Lainie Rutkow

**Affiliations:** ^1^ Associate professor in the Department of Environmental, Agricultural, and Occupational Health at the College of Public Health at the University of Nebraska Medical Center; ^2^ Associate dean for human research protection and a director of the Human Research Protections Program at Johns Hopkins University School of Medicine; ^3^ Associate professor in the Department of Emergency Medicine at Johns Hopkins University School of Medicine; ^4^ Professor and vice provost and chief risk officer at Johns Hopkins University; ^5^ Practice professor in the Department of Health Policy and Management and assistant dean for practice and training at the Bloomberg School of Public Health at Johns Hopkins University; ^6^ Professor in the Department of Health Policy and Management at the Bloomberg School of Public Health at Johns Hopkins University

**Keywords:** human research ethics, institutional review boards (IRBs), infectious disease outbreaks, public health emergencies, humanitarian emergencies, natural disasters

## Abstract

The increasing frequency and severity of various types of societal and humanitarian emergencies—e.g., infectious disease outbreaks and natural disasters—creates unique challenges for conducting human subjects research in these settings. These challenges include regulatory review complexities, maintaining research rigor while increasing efficiency, protecting vulnerable populations, and managing risks. Despite these obstacles, research is critical for improving responses to societal and humanitarian emergencies and protecting public health. Our integrative review examines the use of emergency or rapid response review boards, including ethics reviews and institutional review boards (IRBs), in emergency contexts. An integrative literature search addressed rapid emergency human subjects research reviews across disasters, infectious disease emergencies, humanitarian crises, and public health emergencies. Articles were selected based on pre‐defined criteria, including empirical research, operational action, and theoretical discussion. Data were abstracted and synthesized to identify common themes, gaps, and recommendations. Findings underscore the need for robust IRB support before and during emergencies. Recommendations include pre‐approved protocols, enhanced training, and increased funding and resources for emergency IRB support. Future research should systematically implement these concepts and assess their effectiveness across diverse emergency contexts.

Globalization and climate change have led to an increase in the severity and frequency of infectious disease emergencies, acute humanitarian emergencies, and natural disasters.^1^, ^2^ Human subjects research is challenging to conduct in any environment; in exigent circumstances, the conduct of scientifically rigorous and ethically sound human subjects research can be particularly difficult. Research pursued in settings involving societal emergencies presents unique ethical and operational challenges that may include concerns about benefits to and the vulnerability of the affected population, safety of the research team, the validity of data collected in the emergency setting, and challenges associated with operating in damaged infrastructure and high‐risk environments.^3^, ^4^ Yet, human subjects research can be a critical component of emergency response operations, as it holds the potential to improve future responses and protect the public's health.^5^, ^6^, ^7^

In the wake of natural disasters such as hurricanes and other weather events, for example, there may be an urgent need to study an event's impact on the environment—such as water or air quality—and its impact on the affected human population.^8^, ^9^ In novel pathogen outbreaks, rapid clinical research may facilitate real‐time improvements in the response of public health and other officials, thus enabling evidence‐based clinical decision‐making and promoting rapid implementation of effective public health interventions in environments that otherwise present more questions than answers.^10^, ^11^ Since the onset of the Covid‐19 pandemic, there has been a tidal wave of new research related to the development of diagnostics and screening tools, evaluation of therapeutic and vaccine candidates, and understanding of the pathogenesis of the infection.^12^, ^13^, ^14^ Moreover, research has been conducted on emergency responses to the Covid‐19 pandemic to understand those responses and to inform responses to subsequent infectious disease pandemics.^15^, ^16^, ^17^

Importantly, during times of emergency crises, rigorous research protocols must be maintained.^18^ The need for rapid knowledge acquisition and the push to initiate new clinical trials and other emergency research mechanisms must be balanced against the need to protect research participants.^19^, ^20^, ^21^, ^22^ This requires evaluation of research protocols to identify which results will potentially have the greatest ability to provide meaningful benefit to individuals during emergencies and in the future.^23^ The US federal government has established mechanisms to support rapid human subjects research review, including instantiation of the Public Health Emergency Research Review Board through the US Department of Health and Human Services (as a specialized central institutional review board [IRB] to provide review of public health emergency research conducted or supported by any US executive branch agency or department),^24^ and expedited review of National Institutes of Health intramural research during the Covid‐19 pandemic.^25^ While these two mechanisms can support specific, federally‐funded research, they are not broadly applicable to all emergencies and disasters. Moreover, in natural disasters and acute emergencies, there may not be time to conduct a standard (convened or expedited) review within a typical timeline.

High volumes of emergency research requests require a systematic method at the institutional level for evaluating, prioritizing, and coordinating research efforts to avoid delays in obtaining the approval of an IRB to move forward with human subjects research activities. During a public health emergency or natural disaster, IRB and other review processes should foster the collection of critical human subjects research data in a scientifically rigorous manner that informs later phases of the response and advances clinical management knowledge for future events.^26^ Therefore, for institutional leaders, policy‐makers, and other stakeholders, it is imperative to understand existing evidence on the structure, scope, and potential benefits and limitations of the review by various entities of emergency research protocols.

The goal of this research was to synthesize and critically analyze the quality of the available evidence on the use of emergency or rapid response review boards for human subjects research—including ethics reviews, science reviews, and IRBs—during disasters, infectious disease emergencies, and acute humanitarian emergencies. Given the relatively infrequent use of these emergency reviews, extant empirical research is limited and research methods used to examine this topic are inconsistent. We selected an integrative literature review for its comprehensive nature, which includes incorporation of theoretical and empirical literature and diverse methodologies. Well‐designed integrative literature reviews can enhance evidence‐based practice and provide guidance where limited evidence exists.^27^ This review was conducted to identify and summarize existing evidence related to the use of emergency/rapid human subjects research reviews, document the policies that govern them, assess the type and quality of available evidence, identify gaps in the evidence, and propose additional empirical research requirements to close these gaps.

## STUDY METHODS

We conducted an integrative review of the literature to assess the evidence base for conducting emergency human subjects research reviews. Based on this objective, the investigators identified a series of search terms to systematically identify relevant literature in the PubMed and Embase databases. These databases were selected for their comprehensive biomedical focus and the likelihood of relevant literature being indexed within them. Two databases were used to reduce the likelihood of location bias.

Search terms were designed and refined based on outputs from preliminary searches (see table 1, available online; see information about accessing this material in the “Supporting Information” section at the end of this article). The lead investigator (LMS), with the support of a research librarian, designed the initial search strategy. The terms were identified and expanded or grouped as appropriate. MESH terms—medical subject headings used for indexing articles in PubMed—were used wherever available. Once the search strategy was set, a search string was designed for PubMED and Embase. A secondary review of the grey literature was designed by the lead investigator and adjusted for improved search results by the research librarian.

Articles were included if they were written in English and addressed the use of rapid human subjects research review in disasters, infectious disease emergencies, humanitarian emergencies, and public health emergencies. Articles focused on empirical research, operational action, or theoretical discussion were included.

Articles were excluded if they did not address the use of human subjects research review in disasters, infectious disease emergencies, humanitarian emergencies, or public health emergencies. Articles were excluded if they did not include theoretical discussion or present findings from operational actions or were not empirical research.

The resulting article titles were screened by two investigators (LMS and MKS) independently to determine whether they met inclusion criteria. Results were compared, and any disagreements were adjudicated through a discussion‐based, consensus approach. During this process, both investigators presented their rationale for inclusion or exclusion to one another with the individual who identified the article for inclusion presenting their rationale first.

The same two investigators independently reviewed the abstracts of the remaining articles to determine whether they met the inclusion criteria. Results were again compared and adjudicated with the same process used during the title review. A third investigator (LR) was available to review and adjudicate abstracts for which the two reviewing investigators could not reach consensus.

One investigator (LMS) reviewed all remaining articles in their entirety. Upon full review, articles that the investigator determined did not meet inclusion criteria were excluded. Citations from the works cited/reference list of included articles were reviewed and any potentially relevant articles from these lists were also reviewed for inclusion.

During the process of data familiarization, the following data were abstracted by one author (LMS) from all included articles.

- Geographic location
- Study objectives
- Study design (theoretical or empirical)
- Methodologic approach
- Data source (if any)
- Relevant findings
- Limitations
- Relevance to practice

Abstracted data were synthesized using a qualitative, inductive approach to identify common themes, recommendations, deviation in practice or recommendations, notable gaps, and relevance to practice. The thematic analysis was conducted by one author (LMS)

**The exceptional onslaught of emergency research conducted by institutions during the Covid‐19 pandemic brought to light an urgent need for change.**

in iterative consultation with a second author (LR). A codebook was developed and maintained using NVivo software and inductive coding occurred at both the semantic (explicit) and latent (underlying) levels to elicit emerging themes. This approach enabled the identification of common, explicitly stated findings and deeper conceptual patterns.

The process began with multiple readings of each article as a familiarization process to inform preliminary codebook developments. Initial codes were generated systematically by identifying meaningful segments of text relevant to the research objectives, with particular attention to study findings, recommendations, methodological approaches, and contextual factors.

The codebook was iterated and once finalized, each article was coded in its entirety and emerging themes were continually reviewed for coherence and accuracy by the primary author. An audit trail was created to allow the second author (LR) to review and adjudicate issues with theme development.

## STUDY RESULTS

In total, through searches conducted from January 2020 to June 2023, 11,501 articles were identified. After deduplication, 2,715 articles remained and were screened during title review, leaving 76 articles for abstract review. Of these, 45 articles were excluded during abstract review, leaving 31 for full‐text review. Of these manuscripts, 17 were excluded on full‐text review and 14 articles were included for data abstraction. Thirty‐one additional articles were identified and screened for possible inclusion from a review of the references from full‐text articles. None of these were included in the final set of articles (see figure 1, online).

Fourteen articles were included in the integrative review. Of these, six were descriptive analyses, four were empirical research, three were expert panel reports, and one was a landscape analysis with a proposed framework. The study design, geographic location, study objectives, methodologic approach, and relevant key findings/recommendations are summarized in table 2 (online).

In this section, we present a comprehensive analysis of the findings, focusing on three main areas: administrative preparedness, rigor of rapid reviews, and education and training. This analysis allows for an in‐depth exploration of the operational aspects of human research protections review and how this may be impacted by or during emergencies. Several of the articles are event‐specific, but many are generalized, reflecting the evolution of research ethics in response to emergent threats. Of note, the articles we reviewed focused on US IRBs and/or ethics review committees in other countries, which are called research ethics committees (RECs) or ethics review committees (ERCs). When describing the analysis or findings of an article we reviewed, we used the term the author(s) used in their article. Elsewhere in the paper we use the term IRB to refer to IRBs, RECs, and ERCs, unless there is a specific reason to clarify that we are referring specifically to IRBs in the US.

## ADMINISTRATIVE PREPAREDNESS

Administrative preparedness is essential for ensuring that IRBs can respond effectively to emergencies without having to adjust policies and procedures in real time, potentially risking study quality and protection of human participants. Several articles emphasized the importance of establishing preparatory measures prior to a crisis to ensure integrity of the review process.

O'Mathúna^28^ highlighted the unique ethical challenges of disaster research, advocating for the development of frameworks that adapt general ethical principles to disaster settings. These frameworks should be established in advance of an emergency, and should ensure participant protection and minimize harm, while addressing the urgency of disaster research. Hunt et al.^29^ underscored the need for timeliness, responsiveness, and rigorousness in disaster research protocols. The study detailed strategies employed by RECs, such as ad hoc meetings and email reviews, to expedite the review process while maintaining high ethical standards.

Alirol et al.^30^ described the WHO Ethics Review Committee's experience during the Ebola outbreak, recommending the use of templated materials and fostering collaborative environments to facilitate efficient reviews. This approach enabled the review of protocols within six days of submission, emphasizing the importance of complete and consistent submissions. Aarons^31^ proposed the establishment of a regional ad hoc REC specifically for emergencies in the Caribbean. This model, involving representatives from main RECs, the affected Ministry of Health, and community members, aims to expedite ethics review through enhanced collaboration and coordination.

Packenham et al.^3^ provided 15 recommendations from a working group of the US National Institute of Environmental Health Sciences, focusing on pre‐approved generic protocols and proactive engagement with the disaster research community. Several of these recommendations aimed to balance the need for timely research with the ethical obligation to protect participants and focused on preparedness programming as a tool to ensure readiness. Saxena et al.^32^ described the importance of pre‐developed standard operating procedures (SOPs) for emergency response ethical review, emphasizing the importance of pre‐existing communication and data sharing plans for readiness. Reyes^33^ discussed the REC structure in the Philippines, creating SOPs to ensure rigorous ethics reviews with faster turnaround times during the Covid‐19 pandemic. The study stressed the importance of protocols that ensure review committees are prepared and educated on how to maintain ethical standards while adapting to emergency constraints. Peek et al.^34^ described the successful implementation of pre‐approved IRB protocols and IRB authorization agreements for rapid response disaster research. This case study emphasized the importance of pre‐planning and collaboration among researchers from different disciplines and institutions.

## RIGOR OF RAPID REVIEWS

Maintaining rigor in rapid reviews is crucial for upholding ethical standards and ensuring the integrity of research. Several studies addressed how to balance the need for expedited reviews with thorough and diligent review processes.

O'Mathúna^28^ emphasized that disaster research must uphold the highest ethical standards, suggesting a framework that balances urgency with participant protection and harm minimization. Alirol et al.^30^ highlighted the necessity of thorough ethical review processes even under pressure. They recommended complete and internally consistent protocol submissions to streamline reviews and foster collaboration across different ethics committees.

Lowe et al.^35^ described a rapid response single IRB model implemented during the Covid‐19 pandemic. This model facilitated rapid IRB approval for multisite clinical research, achieving high fidelity review across multiple sites with approval within 72 hours. Key components included streamlined checklists, formal communication plans, and continuous preparedness activities.

Ma et al.^36^ identified specific modifications that could be made to informed consent procedures during the Covid‐19 pandemic as essential for maintaining ethical standards while adapting to emergency constraints. These include the need for modified approaches to informed consent, such as removing the collection of signatures, adapting consent procedures, and using oral consent where appropriate; developing a unified system to ensure the quality of single ERC reviews, which currently does not have a structured or systematic approach; and updating relevant national regulations to make the ERC in China more responsive in an outbreak or pandemic.

Reyes^33^ highlights the need, particularly in low‐resource settings, to avoid therapeutic misconceptions during the informed consent and participation process. They also emphasize the shared responsibility among ethics review committees, researchers, institutions, funding agencies, and regulatory agencies in upholding ethical principles in research at all times. Reyes acknowledges the opportunity to expedite the review process without sacrificing scientific rigor via the use of novel technologies, more frequent meetings, and additional personnel.

Ford et al.^37^ noted that while IRBs across the US managed to review more protocols faster during the pandemic, this was largely due to additional staff efforts. The study highlighted the need for ongoing evaluation of review processes to ensure sustainability and the critical role of expanded personnel that allows for maintenance of quality during an emergency. Ekmekci et al.^38^ identified several challenges faced by RECs during the Covid‐19 pandemic, such as maintaining autonomy, adapting to new regulations, and balancing urgency with ethical rigor. The study called for national guidelines to help RECs navigate these complexities that may cause degradation of rigor in reviews.

## EDUCATION AND TRAINING

Continuous education and training for IRB members and researchers are essential for preparing them to handle the unique challenges of emergency research. Training enhances their ability to conduct thorough reviews under pressure.

O'Mathúna^28^ and Tansey et al.^39^ emphasized the need for training in ethical principles and emergency‐specific review processes. They advocated for adapting ethical guidelines to disaster contexts and ensuring that RECs are equipped to address the unique challenges of emergency research. Alirol et al.^30^ recommended collaborative committee environments that routinely train together to facilitate shared learning and rapid information exchange. Their experience during the Ebola outbreak highlighted the essential benefits of this ongoing training and preparedness.

Packenham et al.^3^ highlighted the role of training in ensuring that IRB members are prepared for the complexities of disaster research. Their recommendations included developing pre‐event protocols and engaging with researchers and responders before disasters occur. Aarons^31^ proposed annual workshops for regional IRB/REC chairs to foster collaboration and improve communication during emergencies. Saxena et al.^32^ advocated for training in SOPs and data sharing plans to ensure consistency and thoroughness in multicountry research efforts. Lowe et al.^35^ stressed the importance of routine educational programming and conduct of regular exercises to maintain preparedness and ensure rigorous reviews.

## DISCUSSION

The findings of our integrative review underscore the necessity of administrative preparedness, rigorous review processes, and continuous education and training to improve human subjects research protections during emergencies. The results, syntheses, and recommendations emerging from this review provide valuable insights into enhancing the capacity and effectiveness of IRBs/RECs/ERCs in responding to crises while maintaining high ethical standards.

### Funding and resource limitations.

Funding and resource limitations are significant barriers to the effectiveness of IRBs during emergencies. During typical operations, many IRBs operate with limited financial resources and insufficient staffing, which become particularly strained during a surge in volume or rapid need for review in emergency situations. This lack of adequate infrastructure can make it difficult for IRBs to rapidly expand capacity to manage increased workload efficiently. The experiences of IRBs from the Covid‐19 pandemic and findings from the studies identified in our integrative review underscore the critical need for enhanced sustained funding and resources to build and maintain robust infrastructure capable of handling the increased workload created by an emergency.

In the current resource‐constrained health care and clinical research environment, additional financial and resource burdens can be a substantial barrier to making any recommended improvements to IRBs. Costs associated with training and education, additional personnel, and outreach were not described in any of the identified articles. While the costs of IRBs in the US are considered an essential expenditure for “indirect cost” funds from grants and contracts, the additional resources required for emergency review processes are not well defined. The resources required for any recommended pre‐work—such as meetings with potential investigators, templated pre‐reviewed protocols, and pre‐event reliance agreements as well as preparedness activities such as trainings, drills, and exercises—are not generally considered in the fee structures of IRBs. Further, maintaining rigor while significantly enhancing the speed at which the review is conducted—or increasing the volume of reviews—creates an additional and unique resource burden.

One recommendation to address these challenges is to advocate for increased financial support to ensure that preparedness is built into the infrastructure of IRBs. This funding could be directed toward expanding IRB staffing, enhancing technological infrastructure to improve the efficiency of reviews, expanding remote staffing during times of surge, and providing training resources focused on how to scale up emergency reviews. Advocacy would be required at both the national and institutional levels, given that most of the costs of US IRBs are supported by federally funded research dollars (including indirect costs charged to funders by institutions). For most institutional IRBs operating within universities and academic medical centers, core operational costs are supported through facilities and administrative indirect cost recovery charged to sponsored research, the majority of which is federally funded.^40^, ^41^, ^42^ As a result, changes to federal research funding levels or indirect cost policies directly affect the operational capacity of these IRBs. IRBs would need to build these requirements into their fee structures so costs are clearly articulated and could be planned for.

To enhance staffing during emergencies, IRB member rosters could be expanded through streamlined recruitment and training of potential members prior to emergencies. Much like emergency response teams that expand their rosters during response operations, IRBs could recruit from pools of pre‐trained individuals, including those who have recently rotated off an IRB, those who have retired but maintained affiliations with their institutions, and/or pre‐trained subject matter experts. These individuals could be trained for times of surging protocol review needs, with additional just‐in‐time training to ensure readiness and the promise of financial incentives if they are called to serve. This approach not only improves an IRB's ability to handle increased workloads but also could increase the breadth of an IRB's technical expertise (if certain subject matter expertise or experience were needed and specifically recruited for ad hoc rostering), ultimately enhancing the protection of research participants and the quality of emergency research. Without resources, however, actions to ensure IRB preparedness in advance of an emergency are unlikely to occur, leading to protracted timelines for IRB reviews in emergencies and possible delays in urgent research response.

### Enhanced training for IRBs.

Just‐in‐time training for IRBs, while crucial for emergency preparedness, presents challenges related to adequacy and sustainability. Reactive just‐in‐time training that is not tied to exercises and other preparedness education may be inadequate or inappropriate for the emergency, leaving IRB members unprepared to handle the unique ethical and operational challenges of emergency research. The findings from this review highlight knowledge gaps among IRB members and staff, which can compromise the integrity of reviews and participant protection. This gap can be addressed by the development of evidence‐based, high quality just‐in‐time trainings for IRB personnel.

To sustain effective just‐in‐time training, it is essential to establish a regular cadence for training and tabletop or functional exercises that complement carefully developed just‐in‐time training. Just‐in‐time training modules focused on emergency‐specific challenges, such as modified informed consent procedures and expedited review protocols, would help prepare IRB staff and members to address the specific challenges of conducting emergency reviews within the context of a given emergency.

Emergencies can introduce unique ethical and regulator dilemmas; ideally, these challenges should not be encountered for the first time during an emergency. Training/education as well as exercises can support the ability of IRBs to adapt policies and procedures, address complex challenges created by emergencies, practice iterative reviews on emergency timelines, develop critical partnerships, and teach members how to amend review processes without compromising review quality or rigor. Further, emergency settings, especially in diverse cultural or conflict zones, require a nuanced understanding of local contexts. Just‐in‐time training focused on these environments can better prepare IRB members to account for these nuances, heighten awareness of potential vulnerabilities, and provide strategies to ensure vulnerable groups are protected. IRBs/RECs/ERCs could leverage the support of emergency management offices within their institutions to begin to build appropriate training and exercise programming.

### Creating an all‐hazards preparedness approach.

In addition to resource and knowledge limitations, the potential for an emergency to impact the basic functions of an IRB can exacerbate strain on the regulatory office. An all‐hazards preparedness approach for IRBs is vital to ensure they are equipped to handle a wide range of emergencies. This type of approach would ensure that IRBs are prepared not just for the technical needs of regulatory review, but for the potential impact of a disaster or emergency on their basic functionality. For example, in the US, the Covid‐19 pandemic required remote work functionality essentially overnight.^43^ Natural disasters may physically impact the ability of an IRB to share protocol documents and other materials or access their existing records. To address this, IRBs need comprehensive preparedness plans that consider various emergency scenarios, including natural disasters, pandemics, and bioterrorism.

Developing and maintaining comprehensive all‐hazards preparedness plans is essential. In the US, the Association for the Accreditation of Human Research Protection Programs (AAHRPP) recognizes the need for IRBs to execute an emergency preparedness plan for the protection of human research subjects during emergencies.^44^ AAHRPP has added a new element requiring emergency plans. While relatively basic, this requirement could serve as a framework for IRB policies to ensure readiness. These plans should be internally reviewed annually or following an emergency response event and updated based on lessons learned from past emergencies and emerging threats. The flexibility and adaptability of these plans are crucial, allowing IRBs to quickly adjust their processes and protocols to meet the specific demands of different emergencies. One opportunity to ensure readiness is for AAHRPP to expand the scope of this requirement—possibly offering templated plans so that IRBs may work together, supporting each other in emergencies and sharing best practices.

Furthermore, promoting continuous improvement processes—such as conducting after action reviews and using them to inform preparedness plans as well as engaging with a diverse range of experts and stakeholders in the development and review of preparedness plans—can provide valuable insights and help address blind spots or biases. This approach ensures that preparedness plans remain comprehensive and balanced, covering a wide range of potential hazards.

## LIMITATIONS

Integrative reviews are exceptional tools for understanding the landscape of literature for a given topic and identifying substantive gaps. Because an integrative review captures a wide variety of approaches, methods, and reports—including quantitative and qualitative data as well as theoretical pieces—it does not allow for an aggregated synthesis of data from all identified manuscripts. Another limitation of this study is that, while there is much to be learned from the approaches of individual IRBs, there is no repository for these types of policies and procedures available to researchers. In the US, individual policies may be found on IRB websites or in internal policy manuals, but they are not systematically available or accessible for scanning and inclusion in this type of review.

## CONCLUSION

Emergency and disaster scenarios present unique challenges that demand a more agile IRB system without compromising the protection of human participants or the quality of IRB review or research protocols. By adopting rapid response protocols, utilizing pre‐approved emergency templates, and improving coordination and communication both internally and externally, IRBs can make more timely and informed decisions and be more effective during an emergency. However, as the speed of operations increases, ethical rigor must be maintained—despite the inherent vulnerabilities created by emergencies—to ensure the welfare of research participants.

The exceptional onslaught of emergency research conducted by institutions during the Covid‐19 pandemic brought to light an urgent need for change. The impact of the volume of Covid‐related human subjects research continues to be felt by IRBs. This global crisis created a unique lens to reassess and reform the operational frameworks governing human subjects research during emergencies. As the urgency of the pandemic wanes, the window to implement lasting changes that could fortify a country's preparedness for future public health emergencies closes, compromising the ability to respond effectively to the next crisis.

Future research should explore how these concepts (adopting rapid response protocols, using preapproved templates, and improving coordination/communication) can be implemented systematically and examine their effectiveness across diverse emergency contexts. Qualitative assessments, including interviews with IRB members, emergency responders, and research participants, can provide valuable insights into the practical and ethical dimensions of accelerated IRB operations. Exploring relationships with departments of emergency management may provide IRBs with additional subject matter experts and additional resources necessary for developing comprehensive preparedness plans, and for conducting just‐in‐time training, simulations and tabletop exercises that can be used to simulate emergency scenarios, and after action reviews. Finally, identifying and advocating for funding that expands IRB preparedness activities is key for improving review processes in emergencies, striking a balance between expediency and precision.

## Supporting information

The tables and figure are available in the “Supporting Information” section for the online version of this article and via *Ethics & Human Research*'s “Supporting Information” page: https://www.thehastingscenter.org/supporting-information-ehr/.

Supporting Information

Supporting Information

Supporting Information
