# Supplementary figures and images for: Human Research Protections during Emergencies: An Integrative Review
Source: Ethics Hum Res. 2026 May 4;48(3):17–26. doi: 10.1002/eahr.70009 (PMC13137931; doi:10.1002/eahr.70009)

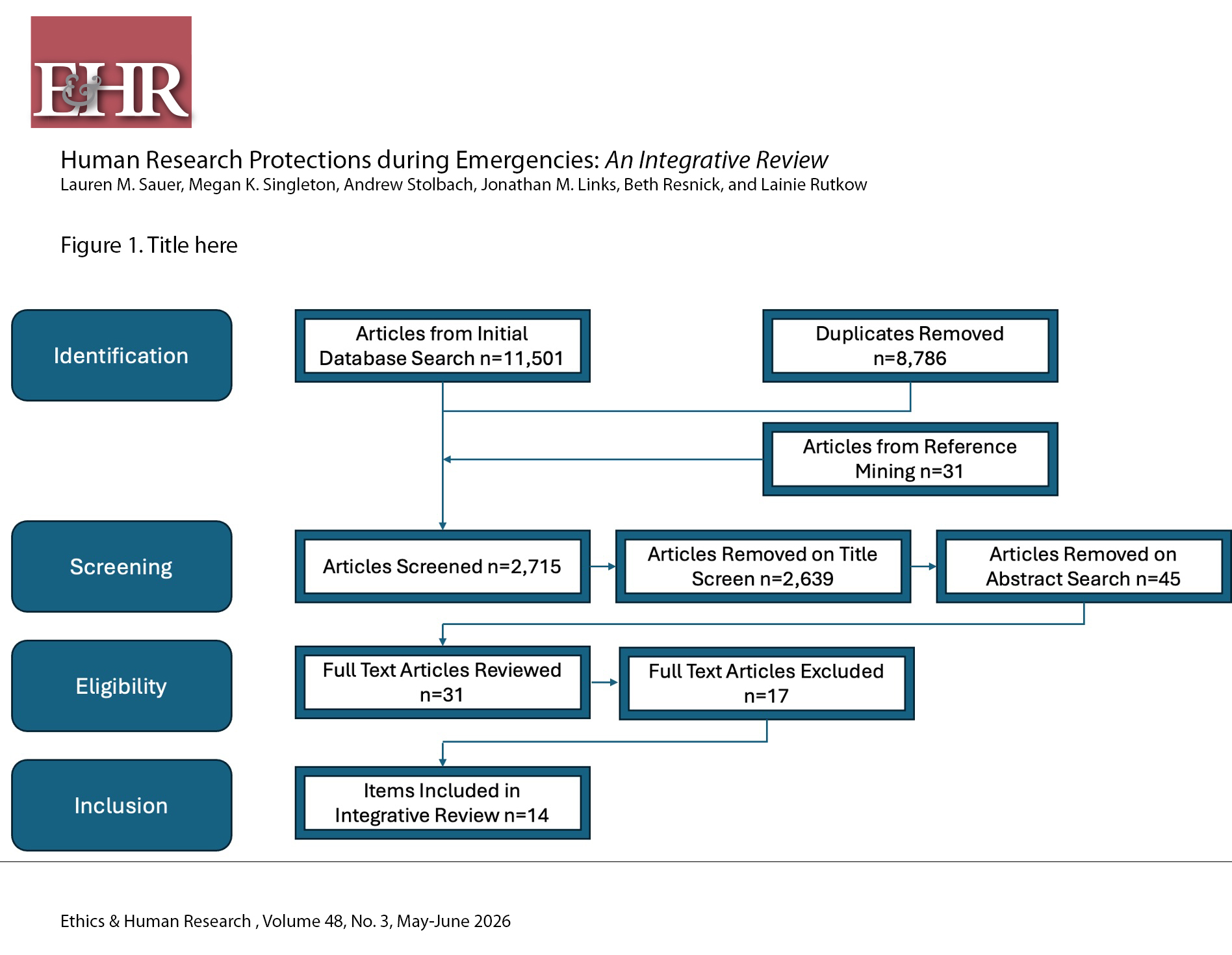

Supplement: Supplementary file 1 — Supporting Information [file EAHR-48-17-s002.jpg]
