# Supplementary material for: Human Research Protections during Emergencies: An Integrative Review
Source: Ethics Hum Res. 2026 May 4;48(3):17–26. doi: 10.1002/eahr.70009 (PMC13137931; doi:10.1002/eahr.70009)
Supplement: Supplementary file 2 — Supporting Information [file EAHR-48-17-s003.pdf]

Human Research Protections during Emergencies: *An Integrative Review*

Lauren M. Sauer, Megan K. Singleton, Andrew Stolbach, Jonathan M. Links, Beth Resnick, and Lainie Rutkow

Ethics & Human Research, Volume 48, No. 3, May-June 2026

E&HR

Table 1: Search Terms and Returned Article Counts

Search Term 1		Search Term 2		# Returned Items
Communicable disease	[mesh]	Ethics Review		2872
		Institutional Review Board	[mesh]	165
		Office of Human Subjects Research Protection		30
		Emergency Research Review		6393
		Scientific Review		3781
Infectious disease	[mesh]	Ethics Review		230
		Institutional Review Board	[mesh]	6
		Office of Human Subjects Research Protection		0
		Emergency Research Review		223
		Scientific Review		594
Disaster		Ethics Review		475
		Institutional Review Board	[mesh]	18
		Office of Human Subjects Research Protection		3
		Emergency Research Review		1624
		Scientific Review		1726
Public health emergency		Ethics Review		2088
		Institutional Review Board	[mesh]	106
		Office of Human Subjects Research Protection		18
		Emergency Research Review		6
		Scientific Review		7105
Emerging Infectious Disease	[mesh]	Ethics Review		29
		Institutional Review Board	[mesh]	1
		Office of Human Subjects Research Protection		0
		Emergency Research Review		223
		Scientific Review		73
Outbreak		Ethics Review		594
		Institutional Review Board	[mesh]	15
		Office of Human Subjects Research Protection		5
		Emergency Research Review		3352
		Scientific Review		3988
Humanitarian emergency		Ethics Review		59
		Institutional Review Board		0
		Office of Human Subjects Research Protection		2
		Emergency Research Review		226
		Scientific Review		93
Pandemic	[mesh]	Ethics Review		467
		Institutional Review Board	[mesh]	15
		Office of Human Subjects Research Protection		2
		Emergency Research Review		1607
		Scientific Review		2990
