# Supplementary material for: Human Research Protections during Emergencies: An Integrative Review
Source: Ethics Hum Res. 2026 May 4;48(3):17–26. doi: 10.1002/eahr.70009 (PMC13137931; doi:10.1002/eahr.70009)
Supplement: Supplementary file 3 — Supporting Information [file EAHR-48-17-s001.pdf]

# Human Research Protections during Emergencies: *An Integrative Review*

LAUREN M. SAUER, MEGAN K. SINGLETON, ANDREW STOLBACH, JONATHAN M. LINKS, BETH RESNICK, AND LAINIE RUTKOW

Ethics & Human Research, Volume 48, No. 3, May-June 2026

Table 2: Summary of Characteristics and Findings from Included Articles

| ID | Author | PMID | Publication Date | Article Type | Research Location | Project Objectives | Methodological Approach | Key Findings/Recommendations |
|---|---|---|---|---|---|---|---|---|
| 1 | O'Mathu'na | 21349047 | 6/2009 | Expert Panel Report | Ireland | Summarize the general ethical issues associated with disaster research and identify distinctive issues that arise in disaster research | Qualitative; in-depth analysis | • Researchers should prioritize the well-being and autonomy of disaster victims and ensure that their research is relevant and beneficial to the affected communities.<br>• The importance of obtaining informed consent from participants and ensuring that confidentiality and privacy are maintained throughout the research process is critical.<br>• Researchers should be transparent about their methods and findings and must consider the potential consequences of their research for all stakeholders involved.<br>• An explicit description of risk and harm reduction is needed in the review to ensure ongoing protection from the risks of the research and the disaster itself. |
| 2 | Tansey | 20530166 | 2010 | Landscape Analysis/ | Canada | Summarize existing ethics review and | Literature Review Policy Review | • Three key elements of a model of research ethics review in emergency circumstances are |

| | | | | | | | | |
|---|---|---|---|---|---|---|---|---|
| | | | | Proposed Framework | | propose new framework | | proportionality, special scrutiny and expedited review.<br>• Proposed model framework for emergency research ethics review highlighting elements of the review that fall into the following categories: Procedural elements that may be altered; procedures that may increase diligence; and, factors relevant to diligence and speed of review. |
| 3 | Hunt | 27327165 | 06/2016 | Original Research | Global | Investigate the experiences and perceptions of individual REC members regarding disaster research review and identify important elements of a high-quality REC review. | Qualitative; in-depth REC member interviews with interpretive description methodology | Specific to RECs reviewing disaster-related protocols in LMICs<br>• 3 main characteristics of high-quality disaster reviews: Timely, Responsive, Rigorous<br>• Assessing whether the research needs to be conducted in the emergency phase is a critical skill/requirement of the REC<br>• Off-the-shelf, pre-reviewed protocols with "in-principle" approvals were recommended by several respondents<br>• Recommendations for 'resisting the pressure of urgency' and maintaining independence |

| | | | | | | | | |
|---|---|---|---|---|---|---|---|---|
| 4 | Alirol | 28651650 | 2017 | Descriptive Summary | Global | Summarize the reviews of the research studies conducted by the WHO-ERC during the 2013-16 Ebola epidemic including interventional (drug, vaccine) and observational studies and create recommendations for improvements. | Descriptive analysis of Ebola emergency reviews by the WHO-ERC and recommendations based on these experiences | • Recommendations focus on investigator-driven pre-work to ensure submissions are appropriate, internally consistent, and complete<br>• generation of pre-emergency template agreements for data and sample sharing<br>• direct information exchange between the chairs of advisory, safety review and ethics committees |
| 5 | Aarons | 28752914 | 2018 | Original Research | Jamaica, St. Lucia | Summarize existing recommendations and produce templates that can facilitate the rapid conducting of research during epidemic or emergency condition0073 | Literature review; qualitative interviews with ministry of health officials, public health practitioners, and research ethics committee/IRB members | Recommended Model:<br>• An 'Ad-hoc' research ethics committee -convened specifically for epidemics and emergencies<br>• Composition: 6 – 7 members (Chairs of the main RECs; Ministry of Health; Community)<br>• Communication: Via Chairs of the RECs back to their committees and institutions<br>• To be implemented under the jurisdiction of the local Ministry of Health<br>• Should convene to familiarize outside of emergency and epidemic settings but should function operationally for |

| | | | | | | | | |
|---|---|---|---|---|---|---|---|---|
| | | | | | | | | epidemic and emergency situations only |
| 6 | Packenham | 28949918 | 2017 | Expert Panel Report | United States | Provide specific recommendations for IRBs that will be tasked with reviewing disaster specific protocols, including engaging the disaster researcher community, and outsourcing disaster research protocols to specialized IRBs | Working group report out using disaster scenarios as prompts; results collated using thematic analysis of WG discussions | Relevant recommendations (rec #):<br>• Increased research risks should not be acceptable in a post-disaster setting. IRBs should ensure clarity and transparency on risks and benefits of research<br>• IRBs should foster conversations/collaboration with the disaster researcher and responder community they are likely to interact with prior to disaster events<br>• Pre-positioned template protocols may be of value to have in place prior to an emergency. IRBs may consider the use of “Contingent Approval” status for time-sensitive disaster studies<br>• Leverage specialized IRBs or designate a specialized IRB for review of disaster-related research to ensure appropriate expertise is in place to conduct the reviews<br>• IRBs should develop templates that will be completed with contextual information |

| | | | | | | | | |
|---|---|---|---|---|---|---|---|---|
| | | | | | | | | regarding the emergency, affected community, study population, and risks to guide IRB review and decision making<br>• IRBs should develop educational programming to foster competency in disaster research; disaster research teams would also benefit from emergency response training |
| 7 | Saxena | 31060618 | 2019 | Expert Panel Report | Global | Identify practical processes and procedures related to ethics review preparedness for low- and middle-income countries based on results from a WHO workshop | Draft recommendations based on plenary sessions and expert panel work in workshop setting; refine recommendations in plenary with experts | Outlines several key recommendations that would facilitate emergency/rapid research review including:<br>• Development and implementation of model SOPs or protocols for communications between NRECs and other key stakeholders<br>• Clarification of the role of the WHO and national level stakeholders in large scale emergencies such as PHEICs where cross-border collaboration is essential<br>• Development of draft/templated REC pre-reviewed sample and data sharing plans in place prior to an emergency |

| | | | | | | | | |
|---|---|---|---|---|---|---|---|---|
| 8 | Ma | 32445288 | 2020 | Descriptive Summary | China | Describe the challenges and obligations of conduct of ethical review committee in the COVID-19 pandemic | Qualitative assessment of ethics review of studies in China conducted during the COVID-19 pandemic using a series of cases to frame potential and actual gaps | Recommendations include:<br>• Adapting approaches to informed consent including removing the collection of signatures, adapting consent procedures, and, using oral consent where appropriate<br>• Develop a unified system to ensure the quality of single ERC reviews<br>• Update relevant national regulations to make the ERC in China more responsive in an outbreak or pandemic |
| 9 | Reyes | 33442166 | 2020 | Descriptive Summary | Philippines | Identify practical processes and procedures for emergency ethics review of research in the Philippines. | Qualitative assessment of existing procedures with recommendations for improved and ethically appropriate rapid review of research in emergencies | Recommendations of relevance include:<br>• limiting emergency clinical trials review to a specific subset of RECs<br>• being prepared to immediately document and report deviations in SOPs and protocols (of review proceedings – not research protocol deviations)<br>• Rapid and structured reporting of challenges for immediate review and adaptation |
| 10 | Sheehy | 33490861 | 2021 | Descriptive Summary | Ireland | Describe the process of establishing a | Qualitive description of a single country's | Key Recommendations include:<br>• Set a targeted scope from onset of the committee to |

| | | | | | | | | |
|---|---|---|---|---|---|---|---|---|
| | | | | | | national approach to research ethics review | approach to establishing a national research ethics review board during the COVID-19 pandemic including barriers experienced and lessons learned. | ensure efficiency and effectiveness.<br>• Build a structure that ensures culture of trust, openness, and integrity across all parties.<br>• Ensure coordinated and open communication channels to foster transparency and successful review processes |
| 11 | Peek | 33960513 | 2021 | Case Studies with Descriptive Summary | United States | Identify improvements in bringing researchers together with the IRB before a disaster to develop plans, procedures, preapproved Institutional Review Board (IRB) protocols and initiate IRB Authorization Agreement (IAA). | Case studies with recommendations that focus both on researcher engagement with the IRBs and federal partners, and opportunities to support IRB-focused preparedness efforts that expedite immediate post-disaster HSR. | Pre-Event specific recommendations include:<br>• Development of a pre-approved “core research protocol” (collaboration between IRB and researchers) that could be updated with the specifics of the disaster, immediately post-event<br>• Leveraging IAAs for federal agencies and academic institutions to be prepared for human subjects research related partnerships prior to an emergency |
| 12 | Ford | 34192061 | 2021 | Original Research | United States | To obtain a holistic view of the rapid protocol activation | Online survey sent to PIs and IRB Directors at Clinical and Translational | Key recommendations/findings:<br>• Designating a single national IRB for responding to pandemics will not likely be |

| | | | | | | | | |
|---|---|---|---|---|---|---|---|---|
| | | | | | | ecosystem that addresses implementation of emergent clinical trials during a pandemic. | Science Awarded Institutions and two follow-on focus groups were conducted for more in-depth information on readiness for a public health emergency | useful and may exclude critical community/local context.<br>• The approach to the expanded requirements and shortened timelines of COVID-19 IRB review was due to an increase in staff time, rather than a reduction in the time a review takes<br>• Recommendation: Add evaluation of how to improve IRB oversight during a pandemic.<br>• Recommendation: Evaluate new approaches to expanding IRB capacity including expanded rosters/review committees, content-specific committees, and distribution of IRB review burden. |
| 13 | Ekmekci | 35150599 | 2022 | Original Research | Turkey | To assess the most important issues impacting ethics committees during the COVID19 pandemic | Online survey developed by the WHO funded Preparedness Planning for Clinical Research During Public Health Emergencies (PREP) group sent to organizations | • Ethics committees should be fully aware of the core values (aligned with global and national norms) that inform their review process and recognize that the unusual circumstances of an emergency may impact them.<br>• The structural approach of the EC may require adaptation during an emergency to |

| | | | | | | | | |
|---|---|---|---|---|---|---|---|---|
| | | | | | | | conducting HSR research during the pandemic | facilitate effective prioritization and effective time-management.<br>• Improved coordination and communication across ECs should be a priority in a large-scale emergency. |
| 14 | Lowe | 35544310 | 2022 | Descriptive Summary | United States | To describe a streamlined, systematic approach to conducting rapid clinical research review using a single-IRB model | Systematic description of the use of an emergency preparedness exercise model for improved ability to test and evaluate the functions of a single-IRB model in an emergency response setting. | • Exercises and exercise evaluation is fundamental to the ability to respond in an emergency and are valuable tools in IRBs preparedness toolkits.<br>• Homeland Security Exercise and Evaluation Program may provide IRBs with the tools necessary to develop exercise models for research response.<br>• Clear points of contact are essential for expediency and coordination using the sIRB rapid response model. |
